# Continuous Monitoring of Recruits During Military Basic Training to Mitigate Attrition

**DOI:** 10.3390/s25061828

**Published:** 2025-03-14

**Authors:** Robbe Decorte, Jelle Vanhaeverbeke, Sarah VanDen Berghe, Maarten Slembrouck, Steven Verstockt

**Affiliations:** 1 IDLab, Ghent University-Imec, Technologiepark-Zwijnaarde 122, 9052 Ghent, Belgiumsteven.verstockt@ugent.be (S.V.); 2 Department of Rehabilitation Sciences, Ghent University, C. Heymanslaan 10, 9000 Ghent, Belgium

**Keywords:** continuous monitoring, military training, attrition, readiness-to-perform, smartwatch, AI, data collection, risk prediction

## Abstract

This paper explores the use of wearable technology (Garmin Fenix 7) to monitor physiological and psychological factors contributing to attrition during basic military training. Attrition, or the voluntary departure of recruits from the military, often results from physical and psychological challenges, such as fatigue, injury, and stress, which lead to significant costs for the military. To better understand and mitigate attrition, we designed and implemented a comprehensive and continuous data-capturing methodology to monitor 63 recruits during their basic infantry training. It’s optimized for military use by being minimally invasive (for both recruits and operators), preventing data leakage, and being built for scale. We analysed data collected from two test phases, focusing on seven key psychometric and physical features derived from baseline questionnaires and physiological measurements from wearable devices. The preliminary results revealed that recruits at risk of attrition tend to cluster in specific areas of the feature space in both Linear Discriminant Analysis (LDA) and Principal Component Analysis (PCA). Key indicators of attrition included low motivation, low resilience, and a stress mindset. Furthermore, we developed a predictive model using physiological data, such as sleep scores and step counts from Garmin devices, achieving a macro mean absolute error (MAE) of 0.74. This model suggests the potential to reduce the burden of daily wellness questionnaires by relying on continuous, unobtrusive monitoring.

## 1. Introduction

In a military context, training programs usually start with Basic Training. The candidates, who are called recruits, in this context must undergo an extensive and demanding training period which usually lasts for around six months.

During training, the candidates have both theoretical and practical sessions to help them learn the principles of being a soldier. These demanding sessions often cause attrition, a term used to indicate quitting the army and returning to civil life. Through the formation, recruits quit the army because they cannot successfully complete the training period. Recruits quit because of several reasons related to the build-up of fatigue, chronic overuse, injuries, or mental wear down [1,2,3]. Attrition costs the military a lot of money, money that could be invested towards further developing soldiers that remain in the army for a longer time. Because the recruits’ bodies and minds are often pushed to their limits, the program must balance testing the recruits to their fullest potential (in preparation of tough military operations) and preventing drop-out and overtraining.

The use of wearable technology (e.g., smart watches, step counters, smartphones) for daily wellness monitoring has been heavily discussed in the scientific literature. In healthcare, for instance, most studies agree that the emerging and ever improving wearable technology can not only partly automate health monitoring, but also make it continuous and unobtrusive [4,5] as compared to the alternative: questionnaires. However, an important aspect of our manuscript is how a large group could be monitored with minimal interference in their day-to-day operations. For example, in military training, it is not feasible to have dedicated ‘laboratory days’ due to the time-constrained schedules they operate under, nor can there be someone on-site 24/7, as everything must be independently operated. Consequently, for the sensor, we had to find a balance between measurement validity and practicality—selecting a device that was compact enough to capture multiple parameters while still being robust enough to withstand the harsh military environment.

In this paper, we will study the physiological and psychological factors contributing to attrition in the recruitment phase at the infantry division of the Belgian military. We will discuss the data processing architecture and provide preliminary data science insights on centrally collected data from two test phases. In total, 63 recruits were monitored during their basic infantry training in the military base at Arlon, Belgium.

## 2. Related Work

Attrition is a phenomenon that has already been studied in the literature. On the mental side, a study by Wolfe et al. [6] in the U.S. military concluded that people with a premilitary interpersonal trauma were 1.5x more likely to drop out of the recruit training. Further, Molloy et al. [7] focused more on the physical side and stated that today’s youth are less prepared for entry-level physical training compared to their predecessors. For physical testing in the U.S. military, they observed the first-time failure rate increasing from 4% in 2003 to 34% in 2009. In Belgium, where this study was performed, more than 40% of recruits leave the army early. Detecting those that are at risk is of the utmost importance.

In the Netherlands, the attrition rate is just as problematic as in Belgium. Furthermore, Huijzer et al. [8], who collaborate with the Dutch Commando Corps, focus on predicting special forces dropout via explainable machine learning on a set of physical and psychological tests performed prior to the training. They found that both physical and psychological variables were related to dropout. More specifically, a higher score on the 2800 m time, connectedness, and skin folds were most strongly associated with dropping out. A similar baseline testing and analysis is also performed in our study and further discussed in Section 5.1. Compared to their study, which only focused on the baseline testing, we also use these insights in the continuous monitoring during Basic Training, as is further explained in the paper. In their follow-up study, den Hartigh et al. [9] extended the baseline testing with a weekly follow-up of experienced psychological and physical stress, recovery, self-efficacy, and motivation. Their results show that low levels of self-efficacy and motivation are significantly associated with dropout and that the dropout could often be predicted multiple weeks in advance based on the weekly follow-up of this data. In our study, we use similar wellness questionnaires, which were collected on a daily basis. Outlier detection on this data is also used to flag recruits that are at risk.

Baseline testing and wellness questionnaires, however, are only part of the puzzle. Combining them with the continuous monitoring of the recruits’ internal/external load, their sleep, and stress gives us much richer information to build models with. A recent study on U.S. Navy populations demonstrated the feasibility of using commercial wearable devices, such as the Oura Ring and ReadiBand, to monitor sleep–wake patterns in operational environments. With over 10,000 person-days of data collected from 845 sailors across multiple ship cohorts (with a respective prevalence rate of 69% and 71% for the ring and band), the study supports the integration of wearables into fatigue management systems, reinforcing the value of continuous monitoring in high-performance settings [10].

For daily health and fitness monitoring of people, the sports and medical world is usually a good example for effective strategies, mechanisms and hardware. A study by Abuwarda et al. [11] researched the possible crossovers of wearable monitoring in healthcare to construction workers. They defined four specific hazards that construction workers are exposed to: slips and falls, collisions with others or materials, stress and fatigue and other factors (e.g., fire and noise). These hazards can be closely monitored by wearables using the sensors equipped on the devices (e.g., accelerometers, body temperature, heart rate, respiratory and location sensors). One must also consider how invasive all these sensors are and what the impact is when multiple of them are worn. Take for example heart rate, which is typically measured with a chest strap. Alternatively, a photoplethysmograph sensor could be utilized. It is a common principle that measures the light absorption of local tissue over time. The principle is often used in wearables to measure heart rate as the amount of light absorption is related to the heartbeat pattern. Heart rate and heart rate variability (HRV) are often used to monitor performance in sports and other physical activities (as reviewed by Stephenson et al. [12] in military context, or by Addleman et al. [13] in sports related fields).

In a publication of Kutilek et al. [14], a review study of the possibilities of wearable technology for the health monitoring of soldiers was performed. In their work, the authors summarized wearable technology that is suited for military use. They conclude that heart rate, electrocardiogram (ECG), and respiration rate are the basic indicators for overall condition monitoring.

In the U.S. Marines, Saxon et al. [15] experimented with Apple iPhones and Watches to continuously monitor the specialized training phase of the elite military units to study how and when attrition takes place. They concluded that most of the withdrawals (23.5%) already took place before day seven of the training. Furthermore, they divided the attrition into different subcategories (i.e., Drop-On-Request, Medical, Safety, Performance). Drop-on-request is the most common one, in which the failure is trainee-initiated. The study used continuous monitoring to better isolate the attributing factors of attrition in this category, showing that continuously monitoring mental and physical status during military training is possible.

Although there are already existing studies that use continuous monitoring in the analysis and prediction of attrition, as discussed above, none of them focus on how to efficiently and effectively implement it in a challenging defence context. This exactly is what we have investigated. In what follows, we further explain the need for a scalable, secure monitoring set-up and describe the technological building blocks of our solution and the challenges related to it.

## 3. Methodology

As mentioned in the introduction, the main goal of this research project is to prevent and understand attrition in the military recruitment phase of the Belgian army. To achieve this goal, a preliminary device and technology were studied. For the device, the main consideration was the need for an unobtrusive device with minimal impact on the daily schedules and sessions of the recruits. Furthermore, the battery life of the device was also an important factor. During training sessions, troops often go on-field and perform orienteering, survival, scouting, and protection tasks in remote areas for multiple days, where no reception or mains power is available. This means that the recording device not only needs to have outstanding battery life but also that the offloading of data needs to be as quick and unobtrusive as possible.

**Figure 1:**
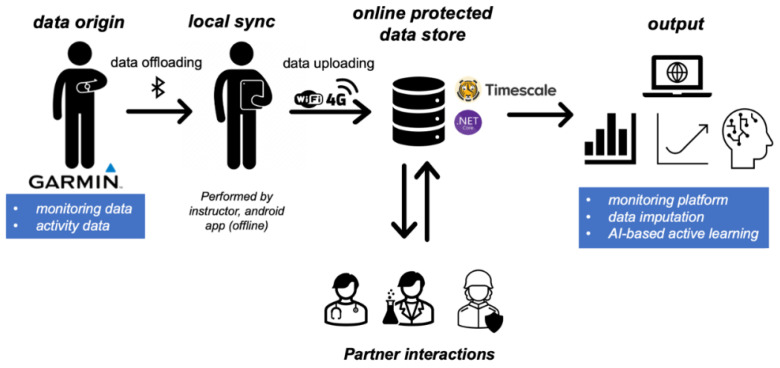
Schematic overview of the data synchronization architecture. Note that an online data store is not a hard requirement as this setup works fully offline as well.

For the data capturing, a central storage solution was developed. This allows for collaboration between researchers and military personnel and enables quick and clean data analysis from a central data source. A schematic overview of the overall data capturing and storage architecture is presented in Figure 1.

The data collection protocol consists of three main elements: the wearable watch capturing health-related monitoring data, a smartphone application to wirelessly collect data from the watches, and a central server to collect all the data from a smartphone.

### 3.1. Wearable Selection

As mentioned in the related work section, Saxon and colleagues proposed a good starting point for monitoring with wearable devices. They used Apple iPhones and Watches to collect the data. This choice of hardware implies frequent device recharging, a task that was assigned to dedicated personnel for their study. For our study, we had the explicit requirement to be as unobtrusive and low impact as possible. This implicated that device autonomy was an important aspect in our final choice. Table 1 shows comparative information between a line-up of two consumer-focused smartwatches and three wearable sensors, which are more often found in heavy-duty industries such as firefighting, military operations, or patient recovery. A big advantage of these consumer-focused smartwatches is that they are more widely applicable for the population and thus more cost-friendly compared to specialized products. Another consideration to make is that with a single device, we can measure a range of physiological parameters, while others (such as the eqLifemonitor and the Axioma Padis) either require multiple attachment points or measure a single parameter. Although these multi-component setups have proven to have a high validity compared to gold standard measurements [16,17], they are still much harder to implement on a large scale with a minimal impact approach (i.e., somebody needs to wear/charge/care for multiple devices).

For our study, we selected a recent iteration of Garmin’s Fenix lineup, the Fenix 7, since it is a balanced combination of an interesting feature set for daily wellness monitoring, user-friendliness, and device sturdiness for a military context (i.e., some watches were severely scratched and damaged on the display after the first testing phase). The device has a claimed 18 days of battery life, with GPS disabled for typical use. For continuous and fine grained location tracking (using GPS, Galileo and GLONASS), the watch can last 40 h, or 57 h when only GPS is used [23]. So, this is a reasonable battery life for expeditions during the training phase when recruits can manually pause location tracking when stationary for prolonged periods of time (e.g., when sleeping or during briefings). The watch is also equipped with useful sensors such as a gyroscope, accelerometer, pulse oximeter, and a wrist-based heart rate monitor. Another very important factor for selecting this device is that for partner institutions, the Garmin ecosystem offers a device management and data extraction Software Development Kit (SDK) for both Android and iOS, called the Health SDK (currently on version 4.2.7). One potential drawback of using a smartwatch as a sensing device is that, in this context, its screen is visible when viewed through night vision goggles. While this issue can be mitigated during training by covering the screen (which also prevents normal watch usage), this may not be a practical solution in operational environments. If the system were to transition to an operational deployment, it might be more appropriate to use the smartwatch primarily for assessing readiness-to-perform rather than having it worn during sensitive night-time missions.

### 3.2. Wearable Management and Data Synchronization

#### 3.2.1. Integrating the Garmin Health SDK

Through the Health SDK, it is possible to take control of the Garmin wearable used in the study through a self created smartphone app that integrates the functionality of the SDK. There are two versions available: the Standard SDK [24] is used to access all health and fitness data through your own services, while the Companion SDK further extends this to access real-time sensor streams. In this research, the Standard SDK was sufficient, as real-time access was not required. So, through our custom app, it was possible to synchronize the watch data while maintaining full control of the data. This is an attractive mechanism in the military since external servers (commonly used to process data, e.g., Garmin Connect servers) can be completely circumvented by use a self provided aggregation/processing server instead. This implies that it can also be deployed in a closed and safe environment, since every aspect is self managed and can even work offline if installed in a local network. In a military context, it is important that all data remain on military network infrastructure so that critical information is not leaked publicly. An extra reason for this choice is that multiple watch connections can be managed on a single phone with the Standard SDK. This means that rather than providing each recruit with a dedicated single syncing device, the data synchronization can be managed centrally and controlled by the study personnel or by one of the commanding officers, which also further reduces the required workload by the military. This principle is further referenced as an operator sync. One downside of the watches is that they are rather costly for use on a large scale. The Garmin ecosystem, however, has lower priced watches (e.g., Venu 2 or Forerunner 245), but often with less battery capacity, a smaller subset of supported features, or lower build quality compared to the Fenix line. Although these watches could be used without modifications to the data syncing and storage strategies, whether all required features are supported should be checked with the Health SDK documentation, for example, checking if on-device sleep analysis is supported. If not, then the SDK will send anonymized data to a Garmin server. To conclude the wearable selection discussion, we collected a user experience questionnaire from the recruits who did wear the watches 24/7 in test phase 1. They reported that they were largely satisfied with the perceived comfort of the chosen watch and no complaints were received during test phase 2.

The watch itself has two important operation modes. Activity mode is equal to that of a watch not managed by the Health SDK and collects relevant data on the selected sport supplemented by location information, etc. This mode is actively initiated and stopped by the recruit or on command by the supervisors. Activities recorded in this mode were based on their itinerary and would often include exercises like orienteering, scouting, and physical education, where the added location information and calculated derivatives are meaningful. On the contrary, when no activity was being recorded, the watch was in monitoring mode. In this case, the watch passively monitored and recorded physiological parameters, such as heart rate, stress, respiration rate, blood oxygen saturation, beat-to-beat intervals, and movement data like the amount of steps and raw accelerometer and gyroscope data. It also collected sleep quality measurements and time spent in sleep stages. Using the management functions of the Health SDK, these functions can be activated on the watch, in addition to the frequency rate at which it should record (for most parameters between 1–3600 s, accelerometer between 1–24 Hz, and gyroscope between 1–32 Hz). Note that the amount spent in activity mode and the amount of functions activated in monitoring mode may have a significant impact on the battery charge and the duration of the synchronization procedure.

#### 3.2.2. Offloading and Processing of the Wearable Data

The offloading of the captured data on the watches to the processing server was performed in two steps. First, the data were collected by the smartphone using the operator sync procedure. Based on the model and the Bluetooth antenna of the smartphone, a fixed number of Bluetooth connections to watches can be maintained. On a Xiaomi Redmi Note 9 Pro and Samsung Galaxy A14, the hardware permits a maximum of 10 open Bluetooth connections be maintained, but a maximum of 5 watches is advised to not saturate the Bluetooth manager (although this can be increased non-concurrently, as further elaborated in Section 5.3). So, using this system, we can achieve at least a 1:5 ratio between the watches and the collection devices, which can be extended to 1:10 through advanced connection management. This is a significant cost reduction compared to a 1:1 sync model. Another option would be to reuse recruits’ personal devices, but this would be much harder to manage. Some control concerning the confidentiality is lost and it relies on the goodwill of the recruits to use everything correctly. It could also create issues about who is responsible for damage to personal devices if they are used for work-related purposes. As an extension, we have also created a parser for watches that are backed up over USB, in which case the data are automatically uploaded to the processing server (and the section below is not applicable).

After the initial pairing of a watch to the smartphone (which only happens once), the watch synchronizes its data to the smartphone. The app and SDK support concurrent synchronization and automatically initiate the data transfer when devices are in range. Whenever new data are received, the app will extract the relevant information out of the transferred binary files before saving to the app’s embedded database. After all the watches are synced to the phone, the operator can initiate the upload process. This will save chunks of data from the internal database to files on the phone’s file system. When a connection to the processing server is available, a request is sent for each created chunk file. This is performed as as to not transfer too many data at the same time (and to respect the http message limits imposed by the server). As a connection is not always stable or may be unavailable due to poor data reception on the field, the app has to contain a failover strategy. So, if no connection is available or it suddenly drops, the chunk files remain on the phone’s file system. The next time an upload attempt is made with a connection, these files will be picked up for transfer first. Moving the data out of the internal database is a necessary step. Based on which measurements are collected (and their sample rate), the app may have to process large amounts of data. By transferring it to outside storage, the queries performed on its database remain fast and thus this keeps the app more responsive. This also makes it less susceptible to memory issues.

**Figure 2:**
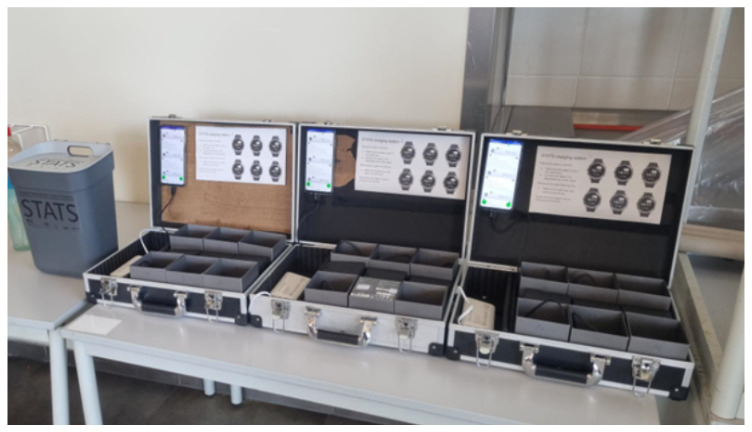
The suitcases used to perform simultaneous charging and synchronization with the smartphones (attached to the suitcases).

During testing, the data were usually uploaded to servers once a week either by study personnel or by one of the commanders of the platoon. The overall syncing strategy can be executed by the military personnel as it is a straightforward process that can be easily performed during, for instance, lunch or a theory lecture. The watches have an advantageous feature in that can sync and charge simultaneously. This can be used to further limit the time spent off a recruit’s wrist. For syncing and charging, we utilized a self-made charging station consisting of a USB charging hub to place and charge nine watches and an attachment point for the syncing smartphone (see Figure 2).

### 3.3. Data Processing

The data on the server were stored in a Postgres SQL (v17.0) database with TimescaleDB (v2.17.0) [25] extensions to allow for efficient date time and timestamp operations. The data were pushed from the smartphones to the server through an Application Programming Interface (API) built with ASP.NET Core (v9.0). This API was also used to manage partner interactions built on top of the collected data. These are outside the scope of this publication, but some examples are as follows: monitoring of the metabolic energy balance [26], analyses of structured running workouts [27] during PE sessions, body scans [28], and the integration of sweat-lactate measurements [29] for selected exercises. Authorized partners can query the information relevant to them and also add their own results in the centralized database. The data on the server were roughly subdivided into five categories: monitoring data, activity data, sleep data, injury reports, and wellness questionnaire data. Monitoring data, activity data, and sleep data are pushed from the watches to the database through the Android application. The other data (i.e., wellness questionnaires and injury reports) can be inserted via a web-based front-end application by the medical examiner (for injury reports) or by the recruits or study personnel for wellness questionnaires (when they are filled out on paper while in a connectionless environment). This workflow allows for the further integration of other measurement tools and information with minimal modification to the base code (e.g., inclusion of blood sample results, clinical refractometer, or nutrition data).

To conclude the discussion on the hardware and software setup, we should mention that the architecture was built with the information criticality and sensitivity of a military context in mind. Firstly, the choice for the Garmin Standard SDK made it possible to keep the data on military-owned hardware (i.e., watches and smartphones), rather than sending the data through Garmin servers (i.e., the Garmin Connect ecosystem). Secondly, the server that the data are ultimately pushed to is also in a protected environment and is managed by the study and military personnel. Both choices imply that with the correct system and hardware security precautions, the data will never leave the military network and thus will not be pushed to the internet or public servers.

## 4. Dataset

Research was conducted during two test phases during 2023 and 2024. A summary of the two studies (test phase 1 and 2) is shown in Table 2a. Test phase 1 lasted 8 weeks, while test phase 2 lasted 17 weeks. The number of recruits, aged between 18 and 27, that were monitored with watches also increased significantly from 17 to 46. The attrition number increased both in absolute values as well as relatively: 18% and 24% for test phase 1 and 2, respectively. Within the attrition numbers, we also investigated reasons for attrition, as reported in the study of Saxon et al. [15] in Table 2b. During the test phases, all attritions were registered under Drop-On-Request or Medical, with the majority being drop-on-request.

Each recruit was equipped with a Garmin Fenix 7 which recorded various types of data. Ranging from full activities with GPS coordinates to passive sensor measurements such as heart rate, steps, stress, body battery, respiration rate, energy expenditure, comprehensive sleep assessments (scores, stages, duration), and beat-to-beat intervals during sleep. Before and during the test phases, additional data were also obtained by means of questionnaires. These questionnaires were divided into two three categories: a series of baseline questionnaires at the beginning of each test phase, a weekly questionnaire, and a daily questionnaire. In test phase 1, these questionnaires were collected on paper using a dedicated mailbox while in test phase 2 used a smartphone app was used to notify recruits to fill in the daily and weekly questionnaires. This change to an electronic collection mechanism was part of an effort to create a more independent and efficient system (compared to pen and paper) and to possibly improve compliance with a more user-friendly interface. Although these changes decreased the workload for the study personnel, we observed a downward trend where the compliance dropped gradually as weeks went by. Efforts by researchers and staff sparked compliance rates for short amounts of time but remained a sticking point unless they were constantly reminded. One of the challenges of this research was to cope with missing data. As later discussed in Section 5.2, we could try to predict missing values using data collected through the watch.

Besides the wearable data and questionnaires, we also collected context metadata from the staff, which gave more insights into the particular courses and sessions the recruits performed. It allowed us to filter the data streams by session and highlighted other information such as the intensity of each session (estimated by the instructor), if they were equipped with heavy equipment or weapons, if it was coupled to an evaluation or other stress-inducing factors, and if it was performed outside. A physical baseline was also obtained for each recruit at the beginning of the test phase, with follow-ups during each phase.

## 5. Results and Discussion

As mentioned in the introduction, the data capturing setup had the goal of finding contributing factors towards attrition and ultimately detecting recruits that might be prone to dropping out the formation in the near future. The results in this paper will mainly focus on two parts: the baseline questionnaire data and the 24/7 monitoring data of the Garmin watches.

### 5.1. Baseline Classification

#### Mental and Physical Baseline

A first analysis was conducted on answers from the questionnaires measuring the psychological characteristics and physical test results of the recruits. Table 3 lists the different questionnaires that were filled in by the recruits. Four published questionnaires were used and a custom domain-specific questionnaire was also conducted. Additionally, the results of the 2400 m running test were also included. The 400 m sprint and 5000 m running test were also measured, but the number of recruits that executed all tests was limited. Therefore, we decided to only include the 2400 m test because the results of the three tests (for the recruits that did all of them) strongly correlated.

Based on eight features (seven psychometric features and one physical feature), we were able to include 56 recruits in the analysis, of which 15 underwent attrition during the course of the Basic Training program. We performed two standard methods: Linear Discriminant Analysis (LDA) and Principle Component Analysis (PCA). LDA is a supervised method that takes into account the classification (attrition or no attrition), while PCA is unsupervised. While LDA performs better, the difference with PCA is small.

Can we identify recruits that are at risk of attrition by solely considering the responses to these questions? In order to find out, we performed supervised dimensionality reduction, where the classification (attrition and no attrition) was used as class labels.

**Figure 3:**
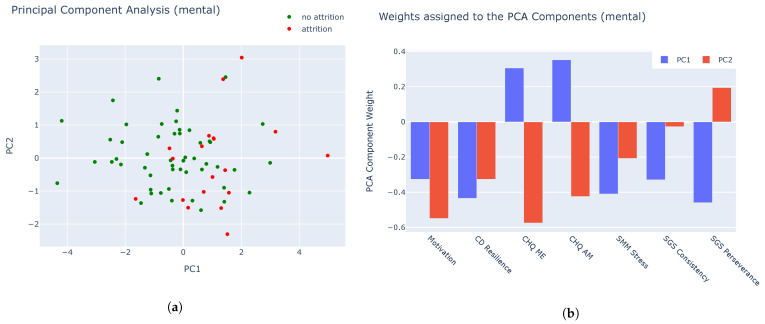
(**a**) PCA based on the answers of the baseline questionnaires (first 2 components). We notice that most of the recruits that underwent attrition are on the right side of the scatter plot. (**b**) Weights of the PCA analysis on the results of the mental baseline questionnaires from Table 3.

**Figure 4:**
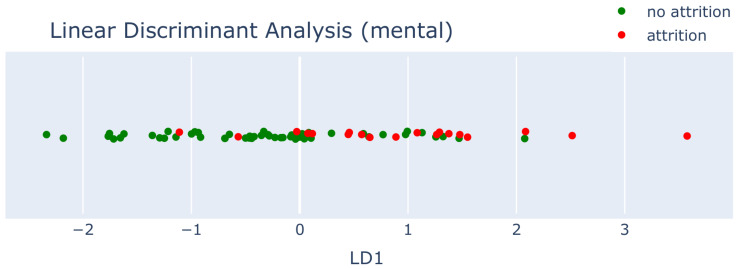
LDA based on the answers of the baseline questionnaires. We notice that most of the recruits that underwent attrition are more frequent for LD1 > 0.

In Figure 3a and Figure 4, we observe that most recruits who experienced attrition are positioned on the right side of the feature space. While distinct clusters are not immediately apparent, this analysis can help identify recruits who may be more susceptible to dropping out. The supervised PCA weights in Figure 3b provide insight into why recruits are positioned on the right side of the graph (PC1 = blue). Both the weights and feature values can be either positive or negative, and to move to the right, their multiplication must yield a positive result. This means that the values must either be both positive or both negative.

**Figure 5:**
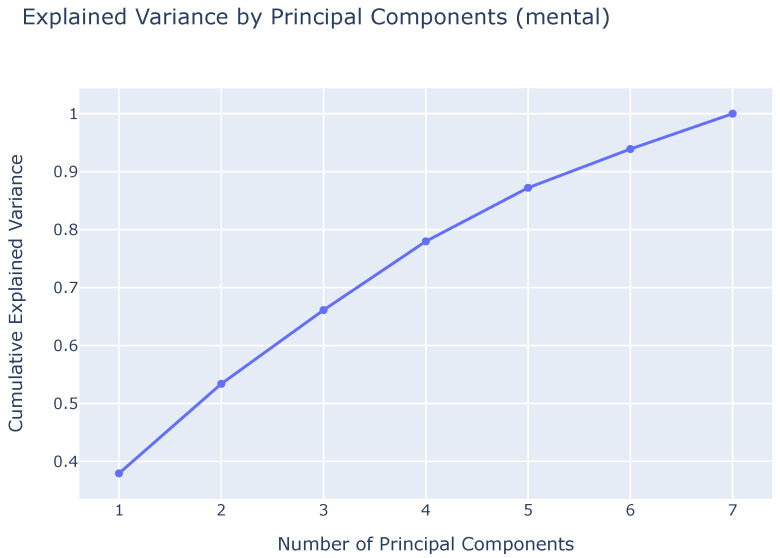
Graph of the cumulative explained variance of the principal components, based on the answers of the seven variables of the mental baseline questionnaire.

By knowing the weights for each feature, we can determine the values that would lead to a recruit moving to the right side of the graph. For instance, a negative weight for motivation means that recruits with low motivation at the start of Basic Training (i.e., a negative value) will push them further along the PC1 axis. Similarly, low scores in resilience (CD Resilience), stress mindset (SMM Stress, which reflects a debilitating stress mindset), consistency (SGS Consistency, which indicates low consistency of interest), and perseverance (SGS Perseverance) will also contribute to an increase along the PC1 axis. On the other hand, CHQ ME (morningness-eveningness) and CHQ AM (amplitude) have positive weights, meaning that higher scores on these traits (which indicate more significant changes in circadian rhythm) will increase the PC1 value. The cumulative explained variance of the principal components is shown in Figure 5.

These results can be used to pay closer attention to recruits with PC1 > 0 and to divide the groups more equally.

### 5.2. Predicting Self-Reported Sleep Scores

Previous studies conducted in different branches of the military of several countries have shown that questionnaire responses can be used to identify at-risk personnel during basic military training [8,34,35]. Although useful, it still causes a disruption when they need to be filled in on a daily basis. Electronic-based systems (e.g., through a smartphone app) require that every recruit has access to such a device and is responsible for it to be charged at the time when the questionnaire should be filled in, which may not always be the case on multi-day operations. In the rural areas where a training camp may be organized, a stable internet connection cannot always be provided. Another approach that uses paper and pen does not suffer from these issues but requires substantially more work and is not scalable since somebody has to input all the forms in the platform, and it is more susceptible to be forgotten since no automated notification can be provided. It also seems to be a step in the different direction when it comes to modernizing data collection protocols in the military. Therefore, it would be useful to develop a method to predict these responses using physiological measurements taken by the Garmin smartwatch. This could alleviate the workload on the recruits by reducing the length of the daily/weekly wellness questionnaires, and perhaps in the future, might eliminate the need to complete the questionnaires altogether. For some cases, it could also be used as a data-imputation strategy, such that missing values in the collected data could be filled in using a predicted value instead of removing that instance.

During the second testing phase, recruits filled in the daily wellness questionnaire (DWQ) every morning during breakfast. It consisted of questions for each of the following categories, which were scored between 1 and 5: sleep quality, motivation, mental recovery, muscle soreness, energy intake, and physical recovery. The total amount of DWQ entries in this test phase was 2543. In the remainder of this section, we focus on developing a predictive scoring technique, focusing mainly on the design for sleep scores with a technique that is transferable to other parts of the questionnaire as well.

We chose to analyse the self-reported sleep scores in greater depth, as this factor is often linked to attrition, particularly in its relationship to stress, fatigue, and motivation. For instance, Taylor et al. [36] found that U.S. Air Force trainees experiencing frequent sleep difficulties were 2.7 times more likely to be discharged compared to those without such difficulties. Similarly, a study by Bulmer et al. [37] revealed that perceived sleep quality had the strongest connection to changes in stress, recovery, and post-sleep fatigue, while the average sleep duration (6.3 h) could negatively affect training outcomes during basic military training (BMT).

**Figure 6:**
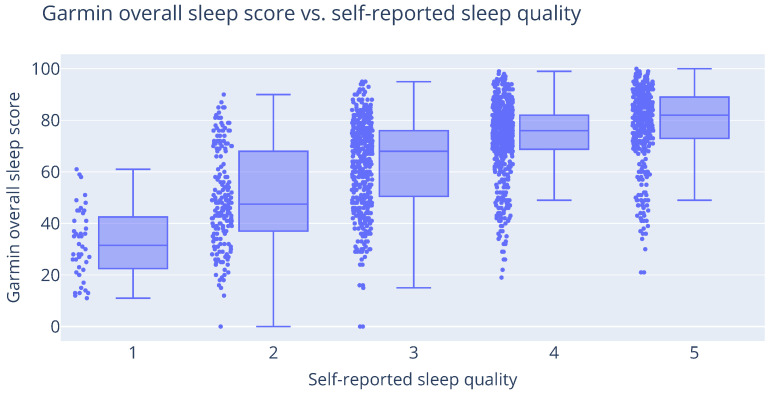
Visualization of the self-reported sleep scores on the questionnaires vs. the score provided by the Garmin wearable. This relationship has a Spearman correlation coefficient of 0.51.

The distribution of each measured sleep score in the function of the reported score is shown in Figure 6. The Spearman correlation coefficient between both values is 0.51. Since this is only a moderate correlation, it is inappropriate to use the reported sleep score as a drop-in replacement for the self-reported sleep score. The (sometimes large) difference could be attributed to the inability to cope with the harsh military environment, which is significantly different compared to the living circumstances of the general population. Some of the error could also be explained by the measurement accuracy of the device. The validity of the hardware itself is not further discussed in this publication, but studies of consumer-focused wearables are still regularly performed [16,17].

As each questionnaire entry was filled in during breakfast, we took the measurements from the day before as the input. Feature extraction of the nightly BBI data was processed using tsflex [38]. Experiments were performed using both strided-window and full-night statistics, but for the technique presented later in this section, the full night measurements were used. From the BBI data, we calculated the following measures: mean, standard deviation, minimum, maximum, maximal difference, and the skew. These are combined with the reported sleep scores of the Garmin of that sleep interval (see Table 4). The final feature used represents the physical exertion level of the day before, represented as the amount of steps taken.

**Figure 7:**
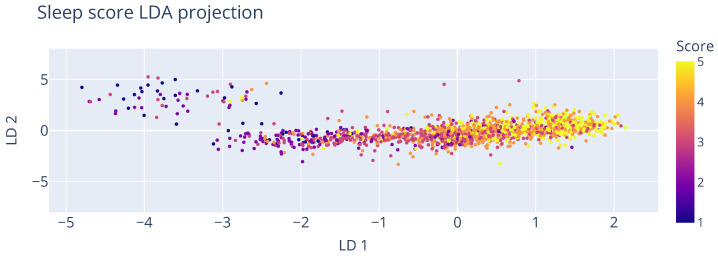
Projecting self-reported sleep scores to the two most discriminative directions using Linear Discriminant Analysis.

**Figure 8:**
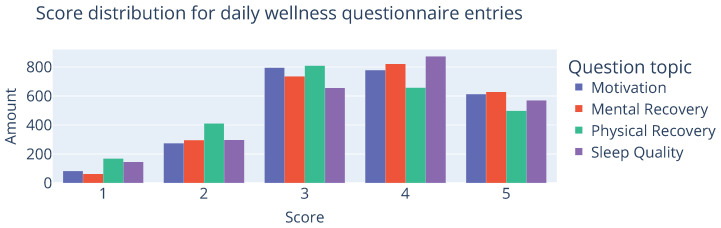
Visualization of the score distributions of the wellness questionnaire entries.

An initial look into the data using Linear Discriminant Analysis (LDA), as shown in Figure 7, indicates that the different scores are somewhat separable using the underlying data. Higher scores of 5 mostly adhere to 0≤LD1, while lower scores are more concentrated at LD1≤−1.5. As this visualization technique only considers a linear relationship, we expect to further refine this with a non-linear modelling technique. However, an important consideration for choosing an appropriate modelling technique is that it has to handle class imbalance well. As shown in Figure 8, the availability of lower scores on the entry distribution is noticeably lower than values in the middle/higher part of the spectrum. This is to be expected due to the fact that most people that will probably feel better more than they feel bad, and those that consistently feel bad are more likely to drop out and no longer provide low values. But since we are mostly interested in these values (as they are the indicators for at-risk recruits), we should take some preventative measures in the design and evaluation of the model to avoid class bias. Therefore, a Balanced Random Forest Classifier (BRFC) [44] was selected for further experiments. The main difference with a traditional random forest is that it takes a bootstrap sample from the minority classes and sample she same number of samples from the majority classes with replacement. This down-sampling technique of the majority class artificially alters the class distribution such that each class is represented equally in each tree.

Evaluation parameters of the model are calculated using 5-fold cross validation. Folds are homogeneous with respect to the recruit, which means that data from a recruit can’t be in the training and the testing set simultaneously. Folds are created by sampling recruits until they contain at least 15% of the recruits. As this is cross-validation, there is no replacement in sampling the recruits. The distribution of each fold is shown in Table 5.

The mean residuals of the predicted scores of the best performing model are shown in Table 6. The macro mean absolute error (MAE) is calculated by averaging the MAEs per class, meaning that the under-represented classes in the splits are not dominated in the overall result. From the different experiments (sets of variables), we concluded that the combination of Garmin scores (from Table 4) and the amount of steps performs best, with an overall macro MAE of 0.74. For comparison, when transforming the steps based on the heart rate zone, the macro MAE was 0.77, and with only Garmin sleep scores it was 0.76; with the sleep scores, BBI derivatives, and steps, respectively, transformed steps, values of 1.05 and 1.11 were found. The feature importance of the BRFC indicates that the sleep duration score (0.21), overall sleep score (0.17), and number of steps (0.13) had the most impact on the predictions. From these results, we conclude that a non-linear transformation on only the Garmin-provided sleep scores can be utilized to estimate self-reported sleep scores (with an average error of 0.74) in harsh military environments.

Table 6 presents the mean residuals of the predicted scores for the best-performing model. The macro mean absolute error (MAE) was computed by averaging the MAEs for each class, ensuring that under-represented classes in the data splits are not dominated in the overall results. Based on the different experiments (using different variable sets), we found that the best performance was achieved using a combination of Garmin sleep scores (from Table 4) and step count, yielding an overall macro MAE of 0.74. For comparison, transforming steps based on heart rate zones resulted in a slightly higher macro MAE of 0.77. Using only Garmin sleep scores produced a macro MAE of 0.76, while incorporating sleep scores, BBI derivatives, and steps (or transformed steps) led to values of 1.05 and 1.11, respectively.

### 5.3. Garmin Fenix 7 Synchronization Benchmark

To ensure smooth integration into the military workflow without adding delays to the already time-constrained schedules of basic training camps, we must focus and optimize two important aspects of the system: simultaneous action execution and concurrent synchronization. The first aspect is already handled by providing the mobile charging stations, such that the data on the watches can be unloaded while charging at an appropriate time (i.e., during a theory or lunch session, such that a minimal amount of activity data are lost). While concurrency is something we can manage through the operator synchronization mechanism. From an operation standpoint, the concurrency factor is mainly limited by the amount of simultaneous connections that can be managed by the BLE manager and the operating system. Through empirical testing and discussions with a Garmin representative, we have learned to limit the number of connected watches to five. While some phones, like the Xiaomi Redmi Note 9 Pro and Samsung Galaxy A14, can support up to 10 connections, exceeding 5 can overwhelm/saturate the BLE manager. This can lead to issues within the Health SDK, resulting in gaps in the transferred data.

From SDK version 4.2.7 onward, new functionality can be leveraged to manage the BLE connections made by the SDK. This means that we are able to programmatically cycle through the paired devices by activating and performing a concurrent sync with a few watches (an insight into how many is given below) before selecting a new set of watches. Since the supported amount of paired but inactive devices is higher than the amount that can be concurrently synced, this approach scales by 2-3x without needing extra phones.

The procedure followed to obtain the benchmark results in Table 7 was as follows. The amount of watches needed for each iteration were loaded with the exact same amount of data, which consisted of two weeks worth of monitoring data. The watches are then paired to a freshly installed app, for instance on a Samsung Galaxy A14. When the app started, it automatically connected with the watches and started the synchronization process. The timings of the start and end signals for each watch were collected through the relevant action handlers provided through the Health SDK. This process was repeated five times for every iteration of the specified amount of watches. Each iteration round in the results table is represented by two measurements, in seconds. The mean individual completion time of a watch (Ind.) is independent of the other watches in the round and the total time required to complete all watches in the round, or the aggregated completion time (Agg.).

This table shows that the variation in completion times for singular devices can be quite large, since the BLE manager takes liberty in the scheduling process of the data transfer and the OS requires extra time/memory for the garbage collector to run. But, compared to the variation in the completion times of the full rounds, it is much lower and thus more consistent. Even though the scheduling behaviour of the BLE manager may seem unpredictable, it has only a limited impact on the total synchronization time. At first glance, without the Bluetooth hopping option, it might appear more efficient to perform one-to-one synchronization five times, as managing multiple concurrent connections adds overheads to the BLE manager. However, scaling this approach comes with significant drawbacks. Either the cost of additional hardware would be paid, or a large amount of manual effort would be needed to ensure only one active connection at a time, such as manually disabling BLE on all other devices and cycling through them. Therefore, the added time required for a more autonomous synchronization process is justified by avoiding these complications. However, most of these problems are irrelevant whenever this connection cycling is performed automatically. In this situation, the only extra delay that must be factored in is the time between sending the reconnection signal and when it actual connects. Typically, this is only 10–20 s, but outliers of 1–2 min have been observed during testing when the Garmin watch prefers battery savings rather than continuously polling for a Bluetooth connection.

Note that the described upper limits of the system may not always be the most performant for every case. By increasing the amount of watches a phone has to cycle through, the required time also increases. In some cases, horizontal scaling may be more appropriate by adding more phones. From our experience with cycling between 10 watches per phone, we conclude that combining the Bluetooth hopping with 2–3 concurrent devices works best.

This gives a general idea of the applicability and time slots in which it should be scheduled in. There are of course other factors that have an impact during deployment and affect synchronization times, such as which activities are recorded (some of which the duration will vary due to it being tied to the effectiveness of the recruit) and if the watch was consistently worn.

## 6. Conclusions and Future Work

In this publication, we present a comprehensive data-capturing methodology designed to monitor recruits during their training phase, alongside preliminary insights from two testing phases. While the current dataset is not yet sufficient to develop early-stage attrition detectors, our analysis identified several potential factors contributing to attrition, including low motivation, resilience, and stress mindset. These findings offer valuable insights for improving the monitoring of at-risk recruits and optimizing group divisions during Basic Training. Follow-up studies are scheduled for late 2024, where we plan to refine both the hardware and software, optimize the monitoring setup, and conduct further analysis into the patterns leading to attrition.

Additionally, we introduced the design of a large-scale, minimally invasive 24/7 monitoring solution aimed at reducing manual intervention during field use. The methodology is still being actively researched to achieve a balance between minimizing the required time and expertise of military personnel and reducing the need for research staff involvement. The long-term goal is to enable autonomous deployment of the monitoring system without research intervention. Future phases will also explore the integration of the wearable device for wellness questionnaires, with the potential to eliminate the need for additional smartphones or paper forms, streamlining the data collection process.

Our analysis of mental and physical characteristics based on eight key features from baseline questionnaires reveals that recruits at risk of attrition tend to cluster on the right side of the feature space in both LDA and PCA. While these clusters have some overlap close to the threshold boundary, they help in identifying recruits more likely to drop out. These insights can better guide support systems for at-risk recruits. Furthermore, our predictive model for self-reported sleep scores, leveraging physiological data from Garmin devices combined with step count, produced promising results, with a macro MAE of 0.74. This method shows potential for reducing the need for and duration of daily wellness questionnaires, particularly in challenging military environments, while still offering reliable insights into recruits’ well-being. Another potential use-case could also be predicting future reports, allowing for the early flagging of potential risk recruits who may require an intervention from the staff.

Future work will focus on refining this predictive approach to further reduce the burden on recruits and to improve accuracy across various wellness categories. Additionally, there is potential to extend this method as a data imputation strategy for filling in missing intervals in the collected dataset, enhancing the robustness of the monitoring system. 

## Figures and Tables

**Table 1:** Comparison between 5 consumer available wearables. Information taken from each respective product/owner brief.

	Garmin Fenix 7 [18]	Fitbit Charge 5 [19]	Movesense [20]	eqLifemonitor [21]	Axioma Padis [22]
**Device type **	Smartwatch	Smartwatch	Wearable sensor	Wearable sensor	Wearable sensor
**Body position **	Wrist	Wrist	Upper arm/chest	Chest	Wrist, belt, backpack
**Price**	EUR 599.99	EUR 150	EUR 104	EUR 1650	EUR 800
**Battery life**	18 days	4 days	16 days HR 7 days ECG	2 days	7 days
**Charging type**	Cable	Cable	Replacable battery	Charging case	Charging case
**Heart rate**	Yes	Yes	Yes	Yes	Yes
**Raw accel.**	Yes *	No	Yes	Yes	Yes
**Raw HRV**	Yes *	Only during sleep	Yes	Yes	No
**ECG**	No	External app	Yes	Yes	No
**GPS support**	Yes	Yes	No	Yes	No
**Synchronization**	WiFi/ANT+/BLE/USB	BLE/USB	BLE ^‡^	BLE/USB	BLE/USB
**Cloud storage**	Yes	Yes	No	Yes ^†^	Yes
**On-premise**	Yes *	No	No	Yes	Yes

* Through usage of the Garmin Health SDK. ^‡^ Can only offload in real time due to the very limited internal storage. ^†^ Only available with an extra service charge.

**Table 2:** Summary of the two on-site test phases. The second test phase lasted longer and monitored more recruits. Attrition is classified into 4 categories: Drop-On-Request, Medical, Safety and Performance.

(a)			
Study	Start date	End date	# recruits
Test phase 1	31 August 2023	27 October 2023	17
Test phase 2	30 January 2024	31 May 2024	46
(**b**)			
**Reason**		**Test phase 1**	**Test phase 2**
Drop-On-Request		3	7
Medical		0	4
Safety		0	0
Performance		0	0
Total		3	11

**Table 3:** Questionnaires used during test phase 1 and 2. Most questionnaires focused on the mental state of the recruits.

Variable	Questionnaire	Reference
Resilience	CD-RISC-25	[30]
Stress mindset	Stress mindset measure	[31]
Sleep	CHR-NL 1–SIF–SIC	[32]
Grit	Short Grit Scale	[33]
Motivation, self-reported health	Custom	
injury, and sports history		

**Table 4:** Overview of the reported sleep scores of the Garmin device. Calculated based on sleep times and sleep stages identified using a combination of heart rate, heart rate variability, pulse oximetry, respiration, and body movement data. Information from this table is directly sourced from different Garmin blogposts [39,40,41].

Measurement	Description
Sleep duration score	How long the recruit slept compared to globally accepted age-based recommendations [42].
Awakenings count score	A high score corresponds to continuous sleep through the night, with few to no stretches of awake time.
Awake time score	Score based on the total time spent awake during the recorded sleep interval.
Interruptions score	Score based on the number of times you are awake for longer than 5 min.
Light sleep score	Score based on time spent in the first stage of sleep. Eye movements and muscle activity slow during light sleep as your body gets ready for deep sleep.
Deep sleep score	Score based on time spent in deep sleep stage. Eye and muscle movements stop completely. Your heart rate and breathing slow. This stage can be referred to as restoration mode, where the body will recover, building bone and muscle, and boosting your immune system.
REM sleep score	Score based on time spent in REM sleep stage. Brain activity is almost as active as when you are awake.
Sleep quality score	Quality aspects of the sleep score come from a combination of sleep architecture, stress data, interruptions during the night and other factors [43].
Sleep recovery score	N/A, no Garmin provided description of this score available.
Sleep restlessness score	This feature indicates sudden movement, typically detected in light sleep.
Overall sleep score	Calculated based on a blend of how long you slept, how well you slept, and evidence of recovery activity occurring in your autonomic nervous system derived from heart rate variability data. This score is calculated on a scale of 0–100 (Excellent: 90–100, Good: 80–89, Fair: 60–79, Poor: Below 60).

**Table 5:** Overview of the data partitioning into 5 unique folds.

	Score 1		Score 2		Score 3		Score 4		Score 5		Recruits	
	Train	Test	Train	Test	Train	Test	Train	Test	Train	Test	Train	Test
Split 1	36	5	152	19	395	47	452	101	259	123	26	7
Split 2	29	12	123	48	330	112	422	131	328	54	26	7
Split 3	33	8	126	45	304	138	434	119	334	48	26	7
Split 4	31	10	131	40	366	76	438	115	305	77	26	7
Split 5	35	6	152	19	373	87	466	87	302	80	28	5

**Table 6:** Residuals of each score over all splits. Macro MAE calculated by averaging all class results in a split. Classwise MAE calculated per score over all splits.

	Score 1	Score 2	Score 3	Score 4	Score 5	Macro MAE
Split 1	0.00	0.53	1.00	1.01	1.21	0.75 ± 0.44
Split 2	0.67	0.94	0.92	0.76	0.61	0.79 ± 0.13
Split 3	0.75	1.02	0.93	0.79	0.48	0.79 ± 0.18
Split 4	0.50	0.90	0.87	0.74	0.73	0.75 ± 0.14
Split 5	0.17	0.90	0.87	0.76	0.53	0.64 ± 0.27
**Classwise MAE**	0.42 ± 0.29	0.86 ± 0.17	0.92 ± 0.05	0.81 ± 0.1	0.71 ± 0.26	

**Table 7:** Synchronization benchmark results for each round of the specified amount of concurrent watches (# Watches). Measured in seconds using the Standard SDK 4.2.3.

# Watches	Round 1		Round 2		Round 3		Round 4		Round 5		Mean Agg.
	Ind.	Agg.	Ind.	Agg.	Ind.	Agg.	Ind.	Agg.	Ind.	Agg.	
1	109	-	128	-	156	-	125	-	123	-	128 ± 15
2	318 ± 7	324	343 ± 15	354	217 ± 6	234	271 ± 12	290	276 ± 36	302	300 ± 40
3	346 ± 52	391	459 ± 31	492	217 ± 69	369	414 ± 18	454	401 ± 41	458	425 ± 45
4	476 ± 181	631	549 ± 31	600	434 ± 184	579	451 ± 162	585	521 ± 51	598	599 ± 18
5	514 ± 216	730	528 ± 200	719	557 ± 151	701	579 ± 290	818	615 ± 161	755	744 ± 41

## Data Availability

Data are not publicly available but may be available from the corresponding author subject to further clarification and subsequent approval from the Belgian Ministry of Defence.
